# NF-κB upregulates glutamine-fructose-6-phosphate transaminase 2 to promote migration in non-small cell lung cancer

**DOI:** 10.1186/s12964-019-0335-5

**Published:** 2019-03-18

**Authors:** Szymon J. Szymura, Jacob P. Zaemes, David F. Allison, Sheena H. Clift, Jaclyn M. D’Innocenzi, Lisa G. Gray, Brian D. McKenna, Benjamin B. Morris, Stefan Bekiranov, Robin D. LeGallo, David R. Jones, Marty W. Mayo

**Affiliations:** 1 Department of Biochemistry & Molecular Genetics, University of Virginia, P.O. Box 800733, Charlottesville, VA 22908 USA; 2 Department of Pathology, University of Virginia, P.O. Box 800733, Charlottesville, VA 22908 USA; 3 Professor & Chief, Thoracic Surgery Service, Memorial Sloan Kettering Cancer Center, 1275 York Avenue, Box 7, New York, NY 10065 USA

**Keywords:** Glutamine-fructose-6-phosphate transaminase 2 (GFPT2), Non-small cell lung cancer (NSCLC), Cell migration, Nuclear factor kappa B (NF-κB), Epithelial-mesenchymal transition, Sirtuin-6 (SIRT6)

## Abstract

**Background:**

Epithelial-to-mesenchymal transition (EMT) results in changes that promote de-differentiation, migration, and invasion in non-small cell lung cancer (NSCLC). While it is recognized that EMT promotes altered energy utilization, identification of metabolic pathways that link EMT with cancer progression is needed. Work presented here indicates that mesenchymal NSCLC upregulates glutamine-fructose-6-phosphate transaminase 2 (GFPT2). GFPT2 is the rate-limiting enzyme in the synthesis of uridine diphosphate *N*-acetylglucosamine (UDP-GlcNAc). UDP-GlcNAc is the obligate activator of O-linked N-acetylglucosamine transferase (OGT).

**Methods:**

Analysis of our transcriptomic data indicates that *GFPT2* is one of the most significantly upregulated metabolic genes in mesenchymal NSCLC. Ectopic GFPT2 expression, as well as gene silencing strategies were used to determine the importance of this metabolic enzyme in regulating EMT-driven processes of cell motility and invasion.

**Results:**

Our work demonstrates that *GFPT2* is transcriptionally upregulated by NF-κB and repressed by the NAD^+^-dependent deacetylase SIRT6. Depletion of *GFPT2* expression in NSCLC highlights its importance in regulating cell migration and invasion during EMT.

**Conclusions:**

Consistent with GFPT2 promoting cancer progression, we find that elevated *GFPT2* expression correlates with poor clinical outcome in NSCLC. Modulation of GFPT2 activity offers a potentially important therapeutic target to combat NSCLC disease progression.

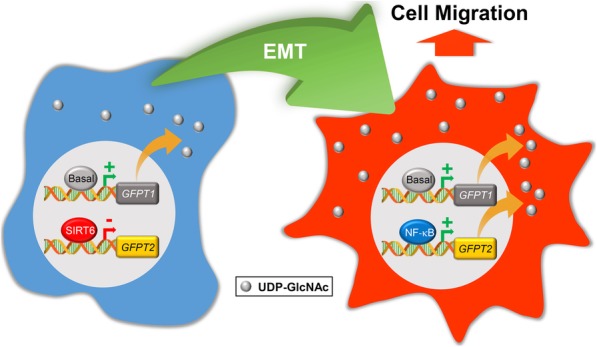

**Electronic supplementary material:**

The online version of this article (10.1186/s12964-019-0335-5) contains supplementary material, which is available to authorized users.

## Background

Lung cancer is the leading cause of cancer-related mortality in the United States and the world [1]. Non-small cell lung cancer (NSCLC) is the most common type of lung cancer, comprised of lung adenocarcinomas (LUAD), squamous cell carcinomas (LUSC), and large cell carcinomas (LULC) [2]. The five-year survival rate for NSCLC is less than 17%, due predominantly to late-stage diagnosis and metastatic dissemination [2]. Therefore, one of the unmet needs is to better understand the molecular processes that govern lung cancer metastasis.

Epithelial-to-mesenchymal transition (EMT) is a physiological process by which epithelial cells lose their cell polarity and cell-to-cell adhesion properties to become migratory [3]. Aberrant activation of EMT in carcinomas initiates cell migratory, invasive and metastatic processes. While numerous growth factors and cytokines present in the tumor microenvironment can initiate the mesenchymal program, one of the best characterized factors is transforming growth factor beta (TGFβ) [4]. Signaling through the TGF receptor activates the SMAD family of transcription factors, which in turn upregulates the EMT master-switch transcription factors TWIST, SNAIL, SIP1, and SLUG. Expression of the master-switch transcription factors initiates epigenetic reprogramming to coordinate differential gene expression associated with mesenchymal phenotypes [5, 6]. Mesenchymal lung cancer cells display dedifferentiated characteristics that are associated with metastatic processes such as increased cell migration, invasion, and cancer stem-like properties [3, 7, 8].

Tumor necrosis factor (TNF) is known to synergize with TGFβ to induce EMT [7–9]. This synergy is due, in part, to the ability of TNF to stimulate nuclear factor kappa B (NF-κB), which upregulates *TWIST1*, *SIP1/ZEB1* and *SLUG/SNAI2* [7]. NF-κB is comprised of five Rel homology domain proteins (RelA/p65, RelB, cRel, p50 and p52) [10]. NF-κB transcription is regulated by the dynamic recruitment of either co-repressor or co-activator complexes to chromatin. Prior to stimulation, p50 or p52 homodimers bind nuclear receptor corepressor (NCoR) or silencing mediator for retinoid and thyroid-hormone receptor (SMRT), tethering class I histone deacetylases (HDAC1, HDAC2, or HDAC3 [11–14]. Upon stimulation the p50/p50 homodimer is de-repressed off chromatin [12–15], and replaced by RelA:p50 heterodimer that recruits coactivator complexes to acetylate RelA at lysine 310 for full NF-κB transcriptional activity [16–18]. Conversely, to actively repress NF-κB transcription RelA:p50 complexes recruit either class I histone deacetylases (HDAC1–3) or the NAD^+^-dependent deacetylases SIRT1 or SIRT6 [11–14, 17, 19].

Highly aggressive carcinomas exhibit elevated glucose and glutamine uptake; two metabolic precursors of the hexosamine biosynthesis pathway (HBP) [20, 21]. HBP synthesizes uridine diphosphate *N*-acetylglucosamine (UDP-GlcNAc); a nucleotide sugar required for the synthesis of glycans and protein glycosylation [22]. UDP-GlcNAc is an obligate activator for the β-*N*-acetylglucosaminyltransferase (OGT) enzyme, which covalently attaches a single O-linked β-*N*-acetylglucosamine (O*-*GlcNAc) moiety to proteins [22]. Several laboratories, including our own, have shown that OGT directly O-GlcNAcylates NF-κB to control its transcriptional activity [23–25].

Although OGT activity is associated with cancer progression [26, 27], the gene targets that link NF-κB with altered cell metabolism and mesenchymal phenotypes remain understudied. Here, we show that TNF and TGFβ stimulation upregulates the HBP rate-limiting enzyme, glutamine-fructose-6-phosphate transaminase 2 (GFPT2). GFPT2 is an NF-κB-regulated gene product that functions to regulate the migratory and invasive properties of NSCLC cells. *GFPT2* mRNA expression correlates with poor clinical outcomes in LUAD. NSCLC tumors exhibit elevated GFPT2 protein expression, linking this metabolic enzyme to EMT and the invasive properties commonly observed in lung carcinomas.

## Methods

### Cell culture and reagents

A549, H358, H1299 NSCLC and HEK293T cell lines were obtained and cultured according to ATCC specifications. Multicellular spheroid cultures were created and stimulated by treatment with TNF (Gibco PHC3016, Gaithersburg, MD, 10 ng/mL) and TGFb1 (Gibco PHG9024, 2 ng/mL) [8]. Knockdowns were performed as previously described [23], using siRNA purchased from Dharmacon (Lafayette, CO, Additional file 1: Table S1). Expression of the non-degradable IκBα supper repressor protein using adenoviral transduction was carried out as described [7]. Doxycycline, puromycin, G418, and Bay 11–7085 were purchased from Sigma-Aldrich (St. Louis, MO). GFPT2 cDNA was obtained from DNASU Plasmid Repository (Tempe, AZ).

### Gene expression and Western blotting

Total RNA was isolated and real-time quantitative polymerase chain reaction (RT-qPCR) analysis was described previously [8] using primers shown in Additional file 1: Table S2, Western blots were performed as described previously [8]. Antibodies used in this study are described in Additional file 1: Table S3, Densitometric analysis was performed on audioradiographs and fold change relative to control samples was calculated using NIH ImageJ 1.46r software [28].

### Metabolic gene analysis

Our previous studies [7, 8] identified 1351 upregulated genes in 3D A549 spheroid cultures stimulated with TNF and TGFβ, compared to unstimulated spheroid controls. Upregulated genes within this list (> 1.5 fold change) were analyzed using BioCyc [29]. Since BioCyc does not include genes encoding for metabolic transport proteins, the list of upregulated genes was also examined for genes encoding the Solute Carrier Family (458 total genes in the human genome).

### ChIP-seq data and GFPT2 gene analysis

ChIP-seq enrichment reads for RelA/p65 performed on TNF-stimulated A549 cells were obtained from GEO series GSE34329 based on data described by [30]. ChIP-seq analysis on A549 cells using histone specific modifications generated by ENCODE/Broad Institute were downloaded from ENCODE (H3K27Ac GEO series GSM1003578, H3K4me1 GEO series GSM1003453, H3K4me2 GEO series GSM1003496, H3K4me3 GEO series GSM1003561, H3K79me2 GEO series, GSM1003512, H3K9Ac GEO series GSM1003544) [31]. ChIP-seq reads were loaded as separate tracks aligned to the human hg19 reference genome and visualized using the Integrated Genome Viewer [32]. ChIP-seq analysis identified two potential RelA/p65 enrichments sites for RelA/p65 binding (Site A and B). DNA sequences encompassing Site A and B (250 base pairs) were analyzed using the MEME suite [33]. Each of the identified Rel cis-elements were then subsequently examined using JASPAR [34].

### Chromatin immunoprecipitation (ChIP)

ChIP assay was performed using a modified Farnham protocol and methods previously described by our laboratory [23]. QPCR ChIP primers used in this study are shown in, Additional file 1: Table S4 (top panel) and were used to analyze the *GFPT2* promoter corresponding to sites A and B (bottom panel, Additional file 1: Table S4). Antibodies used for ChIP assays are listed in, Additional file 1: Table S3.

### Generation of stable cell lines

Stable knockdown of A549 cell lines was generated using doxycycline-inducible shRNA targeting 3′ UTR region of *GFPT2* mRNA and scrambled control shRNA pTripZ vectors (sequences are provided in Additional file 1: Table S1). Lentiviral particles were generated according to manufacturer recommendations (Dharmacon). Virally transduced cells were puromycin selected (1.0 mg/mL), and clonal pools were tested for knockdown efficiency in spheroid cultures treated with TNF and TGFβ. A549:shRNA cells were treated with doxycycline (0.5–1.0 mg/mL) prior to cytokine treatment. Stable H1299 cells overexpressing GFPT2 (H1299:iGFPT2), or luciferase control (H1299:iControl), were generated using the doxycycline-inducible pRetro Tet-On system (Takara Bio, Mountain View, CA). H1299 cells were selected with puromycin and G418 (1.0 mg/mL each) for two weeks. H1299 cultures were doxycycline treated to achieve GFPT2 expression prior to cytokine addition.

### Transwell migration and scratch assays

Migration and Invasion assays were performed as previously described [8] using Boyden Transwell chambers (Corning, Corning, NY). For wound healing assays spheroids were trypsinized and cells were plated near confluency in 24-well plates. A scratch was made in the monolayer using a sterile p200 pipette tip. The next day, the plates were washed with PBS and fresh media with reduced serum (2% FBS) was added to the wells. Gap closure was monitored at 24, 48, and 72 h. Gap surface measurements were done with TScratch software [35].

### Immunohistochemistry

Immunohistochemistry (IHC) was carried out using zinc formalin-fixed paraffin-embedded tissue microarrays (LC121a and LC1921a, US Biomax, Derwood, MD). GFPT2 was detected by IHC using the HPA059910 antibody and tissues were counterstained with hematoxylin. Immunostains were examined in an objective semi-quantitative fashion using criteria previously published [8].

#### Statistics

All data are presented as mean ± SD of three independent biological experiments. Fold changes were log_2_-transformed and a one-tailed Student’s *t* test was performed by using Microsoft Excel. Differences were considered statistically significant when indicated by *p* ≤ 0.05 (indicated by *), *p* ≤ 0.01 (**), or *p* ≤ 0.005 (***). Samples that were not significant (ns) were noted, relative to controls.

## Results

### OGT is required for EMT in A549 cells

**Fig. 1:**
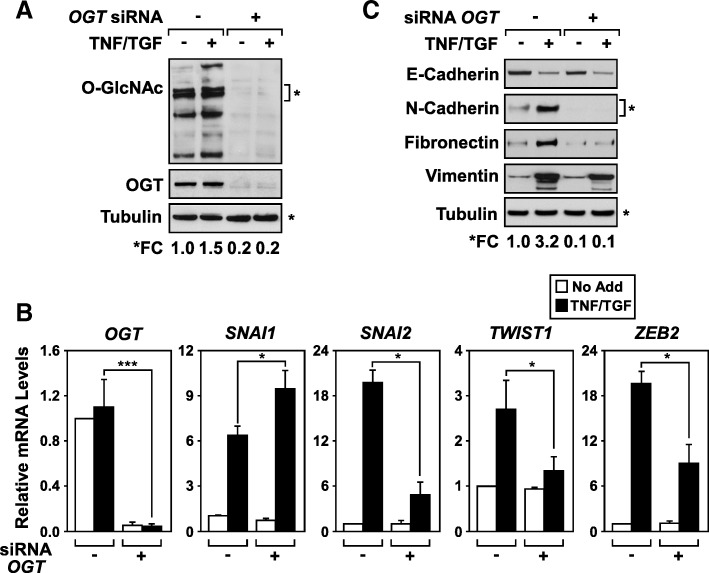
OGT is required for the induction of EMT. A549 cells were transfected with OGT siRNA (+) or control siRNA (−) followed by culturing 3D spheroids with (+) or without (−) the addition of TNF and TGFβ. **a** Immunoblotting of total cell lysates demonstrates that OGT is required for the accumulation of O-GlcNAcylated proteins in cytokine-treated A549 cells. **b** RT-qPCR analysis confirms that OGT expression is required for the cytokine-induced expression of *SNAI2*, *TWIST1* and *ZEB2*. Changes in mRNA expression were calculated relative to *HPRT* with mean and SD + shown; * *p* = < 0.05, ** = < 0.01, and *** < 0.005, *n* = 3. **c** Immunoblotting of total cell lysates demonstrates that OGT is required for cytokine-responsive upregulation of mesenchymal protein markers, N-cadherin, Fibronectin, and Vimentin. **a** & **c** Densitometic quantification of immunoblots demonstrates that the knockdown of OGT significantly dampens global O-GlcNAcylation and suppresses expression of mesenchymal protein markers relative to tubulin. Immunoblots are a representative example from at least three independent experiments

Several groups have reported the requirement of OGT for metastatic properties [26, 27]. To examine whether OGT is required for the induction of EMT in lung adenocarcinoma (LUAD), OGT was knocked down in three dimensional (3D) A549 spheroid cultures [8]. A549 spheroids stimulated with TNF and TGFβ showed an overall increase in global O-GlcNAcylated proteins (Fig. 1a). Knockdown of OGT efficiently silenced OGT expression at both the mRNA and protein level (Fig. 1a and b). As predicted, knockdown of OGT effectively abolished the addition of the O-GlcNAc moiety on proteins at a global level. Silencing of OGT significantly inhibited the gene expression of the EMT master-switch transcription factors (*SNAL2*, *TWIST1*, and *ZEB2*) without altering *SNAL1* expression (Fig. 1b). The knockdown of OGT correlated with the inability of A549 cells to upregulate EMT markers (N-cadherin, fibronectin, and vimentin) without affecting protein expression of the epithelial marker E-Cadherin (Fig. 1c). Collectively, these results corroborate previous findings that OGT activity is associated with the induction and maintenance of EMT [36].

### Mesenchymal NSCLC cells upregulate genes involved in UDP-GlcNAc synthesis

**Fig. 2:**
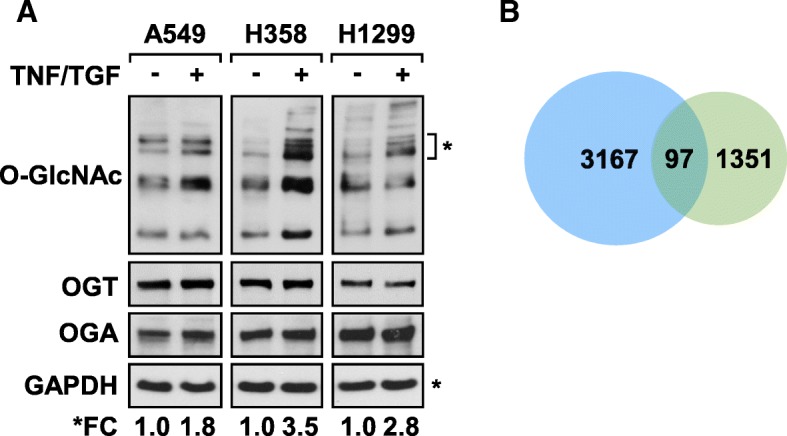
Mesenchymal NSCLC upregulate enzymes involved in the synthesis of UDP-GlcNAc. **a** Immunoblotting of total cell lysates demonstrates an increase in O-GlcNAcylation following TNF and TGFβ stimulation of 3D NSCLC cultures without notable changes in OGT or OGA protein levels. Densitometry analysis of immunoblots were used to determine the fold changes in O-GlcNAc levels relative to the GAPDH loading control. Asterisks mark the bands used for densitometric analysis to calculate the fold change (FC). **b** Venn diagram illustrating the overlap between genes upregulated in mesenchymal A549 cells (green) with the total number of metabolic genes in human genome (blue). Analysis identified 97 metabolic genes specifically upregulated in mesenchymal A549 cells. FDR < 0.05

Cytokine treatment of A549, H358, and H1299 3D cultures resulted in modest, but consistent, global increase in O-GlcNAc modified proteins in cell extracts (Fig. 2a). The increase in global O-GlcNAcylation was not associated with an upregulation of O-linked N-acetylglucosamine transferase (OGT) protein expression or with the loss of O-GlcNAcase (OGA) protein levels in the NSCLC cells analyzed (Fig. 2a). Since UDP-GlcNAc is an obligate activator of OGT [37], our results suggest that the overall increase in global O-GlcNAc levels observed in Fig. 2a is the result of elevated de novo UDP-GlcNAc synthesis increasing OGT activity.

Based on results in Fig. 2a, gene expression data from TNF and TGFβ-stimulated A549 spheroids was analyzed for changes in metabolic genes. Gene expression analysis identified 1351 genes that were transcriptionally upregulated in 3D A549 cultures upon stimulation [6]. This gene list was analyzed for overlap with a previously annotated library of metabolic genes [29], as well as with genes encoding the Solute Carrier Family of proteins. As illustrated in the Venn diagram (Fig. 2b), ninety-seven genes overlapped between differentially upregulated genes in cytokine-treated 3D cultures and the metabolic gene list (Additional file 1: Table S5). Twelve of these genes, with > 1.5-fold increase in expression, contribute to metabolic precursors required for the synthesis of UDP-GlcNAc (Table 1).

**Table 1:** Upregulated Genes Required for UDP-GlcNAc Synthesis

Symbol	Description	GenBank	Fold Change
*GFPT2*	glutamine-fructose-6-phosphate transaminase 2	NM_005110	6.567
*NT5E*	5′-nucleotidase, ecto	NM_002526	3.528
*HKDC1*	hexokinase domain containing 1	NM_025130	3.414
*UPP1*	uridine phosphorylase 1	NM_003364	3.209
*SLC2A1*	solute carrier family 2 (facilitated glucose transporter), member 1	NM_006516	3.003
*SLC2A3*	solute carrier family 2 (facilitated glucose transporter), member 3	NM_006931	2.700
*SLC1A4*	solute carrier family 1 (glutamate/ neutral amino acid transporter), member 4	NM_003038	2.695
*SLC2A6*	solute carrier family 2 (facilitated glucose transporter), member 6	NM_017585	2.672
*HK2*	hexokinase 2	NM_000189	2.555
*PGM2*	phosphoglucomutase 2	NM_018290	1.537
*CDA*	cytidine deaminase	NM_001785	1.515
*UAP1*	UDP-N-acetylglucosamine pyrophosphorylase 1	NM_003115	1.500
			*p* = < 0.05

### *GFPT2* transcripts are upregulated with known mesenchymal genes

**Fig. 3:**
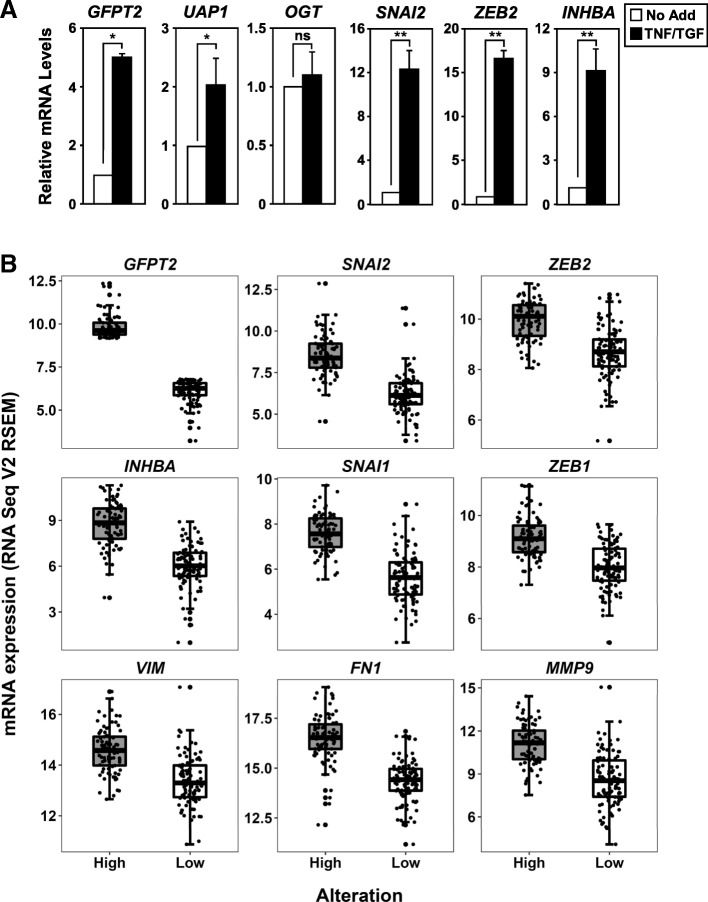
Transcripts encoding *GFPT2* are induced in A549 and co-expressed with known markers of mesenchymal transition. **a** RT-qPCR analysis confirms that two of the enzymes in the HBP pathway, *GFPT2* and *UAP1,* are transcriptionally upregulated in mesenchymal A549 cells, while *OGT* expression remains relatively unchanged. Mesenchymal A549 cells upregulate *GFPT2* along with other known mesenchymal transcripts, *SNAI2*, *ZEB2* and *INHBA*. mRNA expression is represented as fold change compared with the unstimulated control cells with samples normalized to *GAPDH*. Data are calculated mean + SD, * *p* = < 0.05 and ** = < 0.01, *n* = 3. NS - not significant compared with controls. **b** TCGA data (LUAD, 517 samples) were stratified between *GFPT2* high (> 9 RNA Seq V2 RSEM) and low (< 6 RNA Seq V2 RSEM) using cBioPortal [49, 50]. g The mesenchymal gene signatures that are enriched when *GFPT2* expression is elevated are shown and highly significant based on the *p* values, which ranged from 2.11E-03 to 4.23E-12

To validate results shown in Table 1, gene expression levels were analyzed in TNF and TGFβ-stimulated 3D A549 cultures. Mesenchymal A549 cells upregulate genes encoding for glucose transporters (SLC2A1/GLUT1), glutamate transporter (SLC1A4), nucleoside import (NT5E), and pyrimidine nucleoside phosphorylase (UPP1, Additional file 1: Figure S1). Importantly, mesenchymal A549 cells showed a significant increase in transcripts encoding for enzymes that directly contribute to the synthesis of UDP-GlcNAc, glutamine-fructose-6-phosphate transferase 2 (GFPT2) and UDP-N-acetylglucosamine pyrophosphorylase 1 (UAP1, Fig. 3a). GFPT2, the first and rate-limiting enzyme of the HBP, was the highest upregulated metabolic gene identified in our analysis. Since *OGT* gene expression did not significantly change (Figs. 2a and 3a), our results suggest that the increase in global levels of O-GlcNAcylation observed in mesenchymal A549 cells correlates with upregulation of gene products that contribute to the synthesis of UDP-GlcNAc.

*GFPT2* transcripts were upregulated in 3D A549 cultures following TNF and TGFβ stimulation, similar to well-characterized mesenchymal gene targets, *SNAI2* and *ZEB2, and INHBA/Activin A* (Fig. 3a). Our laboratory has previously shown that *INHBA* encodes for an autocrine factor required for cancer stem-cell like properties [8]. LUAD tumors with elevated *GFPT2* mRNA expression displayed significant enrichment of other well-known mesenchymal genes encoding for EMT master-switch transcription factors (*SNAI1*, *SNAI2*, *ZEB1*, and *ZEB2*), as well as *INHBA*, vimentin (*VIM*), fibronectin 1 (*FN1*), and the matrix metallopeptidase 9 (*MMP9*, Fig. 3b). Collectively our data indicates that *GFPT2* is one of the top metabolic genes upregulated in mesenchymal A549 cells and that *GFPT2* is co-expressed with other mesenchymal gene signatures in LUAD based on TCGA data.

### *GFPT2* is an immediate-early gene maintained in mesenchymal LUAD cells

**Fig. 4:**
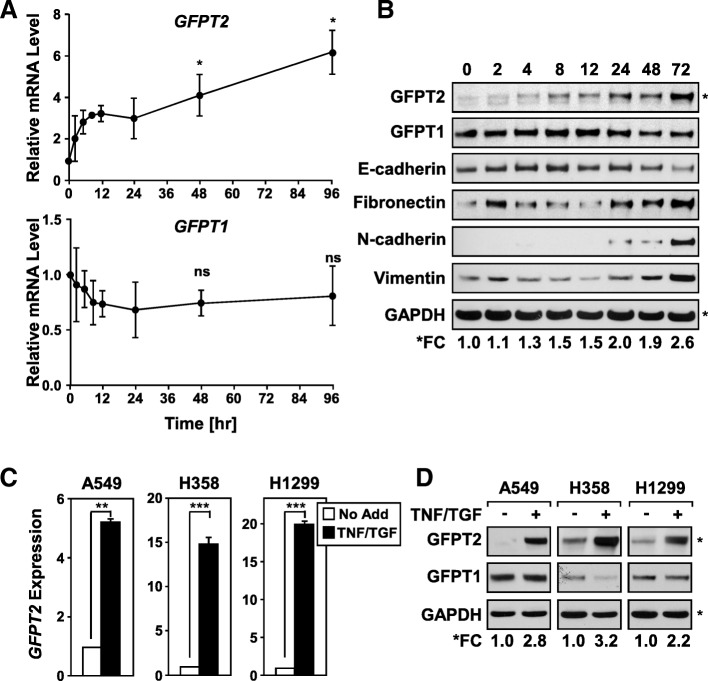
*GFPT2* is an immediate-early gene product expressed in mesenchymal NSCLC cells. **a** Mesenchymal A549 cells upregulate and maintain *GFPT2* mRNA transcripts over a 96 h time course, while *GFPT1* expression remains relatively unchanged. **b** Immunoblot analysis demonstrates an increase in GFPT2 protein expression that corresponds with protein markers of mesenchymal transition. **c** & **d** TNF/TGF-stimulated 3D NSCLC lines upregulate *GFPT2* mRNA and protein expression. **a** & **c** RT-qPCR analysis represents three independent experiments performed in triplicate; mRNA levels were calculated relative to *HPRT* mRNA expression. Mean + SD are shown * *p* = < 0.05, ** = < 0.01, and < 0.005. Immunoblots were analyzed for GAPDH as a loading control (**b)** and (**d)**. Densitometic analysis of immunoblots was used to quantitate fold change of GFPT2 relative to GAPDH

To further characterize the expression profile of *GFPT2*, a ninety-six hour TNF/TGF treatment time course was performed. *GFPT2* mRNA was induced 2 h post-TNF/TGFβ stimulation, and its levels gradually increased and remained elevated throughout the time course (Fig. 4a). In contrast, *GFPT1* mRNA expression showed a modest decrease over the same time frame. Consistent with the observed elevation of *GFPT2* mRNA expression, mesenchymal A549 cells displayed a gradual increase in GFPT2 protein level over the seventy-two-hour time course, compared to GFPT1 (Fig. 4b). GFPT2 protein expression increased with similar kinetics as other established protein markers of mesenchymal transition, namely elevated fibronectin, N-cadherin and vimentin and loss of E-cadherin expression. Similar increases in the transcript and protein expression for *GFPT2* was observed in other NSCLC cell lines H358 and H1299 (Fig. 4c & d). The increase in mRNA and protein expression observed in A549, H358, and H1299 was specific for GFPT2 and was not observed for GFPT1 (Additional file 1: Figure S2).

### GFPT2 is an NF-κB-responsive gene target

**Fig. 5:**
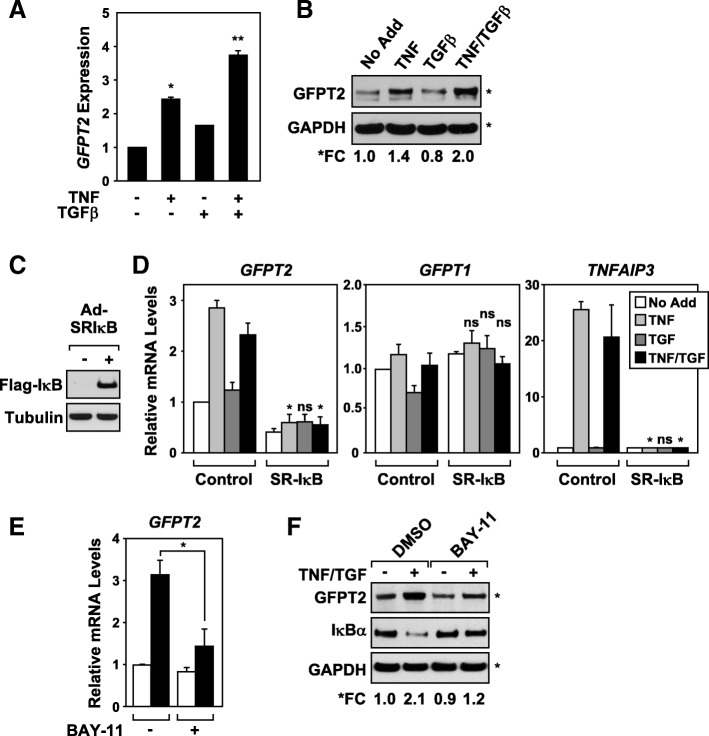
Cytokines upregulate *GFPT2* mRNA expression in an NF-κB-dependent manner. **a** & **b** A549 spheroid cultures upregulate GFPT2 mRNA expression and protein expression in response to TNF, or combination of TNF and TGF. **c** & **d** A549 cells transduced with adenovirus encoding a non-degradable form of the NF-κB inhibitor, SR-IκBα (+) were unable to upregulate *GFPT2* mRNA expression in response to TNF or TNF/TGF, relative to GFP control (−) cells. **e** & **f** A549 cells treated with Bay 11–7085 (5 μM) or vehicle (DMSO) for one hour, prior to a two hour stimulation with TNF/TGF, show that IKK activity was required to increased *GFPT2* mRNA and protein levels. **f** Detection of total IκBα protein levels was used as a measurement of IKK activity. RT-qPCR analysis represents three independent experiments; mRNA levels were calculated relative to *HPRT* mRNA expression. RT-qPCR performed on *GFPT1* and *TNFAIP3* served as negative and positive NF-κB regulated gene targets, respectively. Mean + SD are shown * *p* = < 0.05, and ** = < 0.01; ns = not significant relative to controls. Immunoblots were analyzed for protein loading controls, GAPDH or Tubulin

To determine whether *GFPT2* expression is differentially regulated, A549 spheroids were treated with TNF, TGFβ, or with both cytokines. A549 cells upregulated *GFPT2* transcripts and protein expression in response to TNF, but only modestly in response to TGFβ (Fig. 5a and b). The most robust upregulation of *GFPT2* expression was observed when spheroids were stimulated with both cytokines. To address whether *GFPT2* was transcriptionally regulated by NF-κB, we utilized an adenovirus encoding a non-degradable mutant IκBα protein (super-repressor IκB, SR-IκB) that specifically blocks NF-κB nuclear translocation and transcription [7]. As shown in Fig. 5c, A549 cells effectively expressed the Flag-tagged SR-IκB protein. Consistent with our previous observations, TNF and the combination of TNF and TGFβ, but not TGFβ alone, was able to upregulate *GFPT2* mRNA levels in GFP-expressing control cells 2 h following stimulation (Fig. 5d). Expression of the SR-IκB protein completely abolished TNF-dependent induction of *GFPT2* and decreased basal *GFPT2* mRNA levels (Fig. 5d). Cells expressing SR-IκB failed to show differences in *GFPT1* mRNA expression. We confirmed that the SR-IκB protein effectively blocked NF-κB transcription based on its ability to inhibit TNF-induced expression of *TNFAIP3*, a well-known NF-κB regulated gene [38]. Next, pharmacological inhibition of IKK using Bay 11–7085 demonstrated that IKK/NF-κB was required for TNF/TGF-induced *GFPT2* mRNA and protein expression (Fig. 5e and f). Bay 11–7085 blocked IKK activity as demonstrated by its ability to prevent IκBα degradation following TNF/TGF stimulation, compared to vehicle control (DMSO, Fig. 5f). These observations indicate that NF-κB is required to upregulate *GFPT2* mRNA expression following cytokine stimulation.

### The GFPT2 gene locus is differentially regulated by NF-κB and SIRT6

**Fig. 6:**
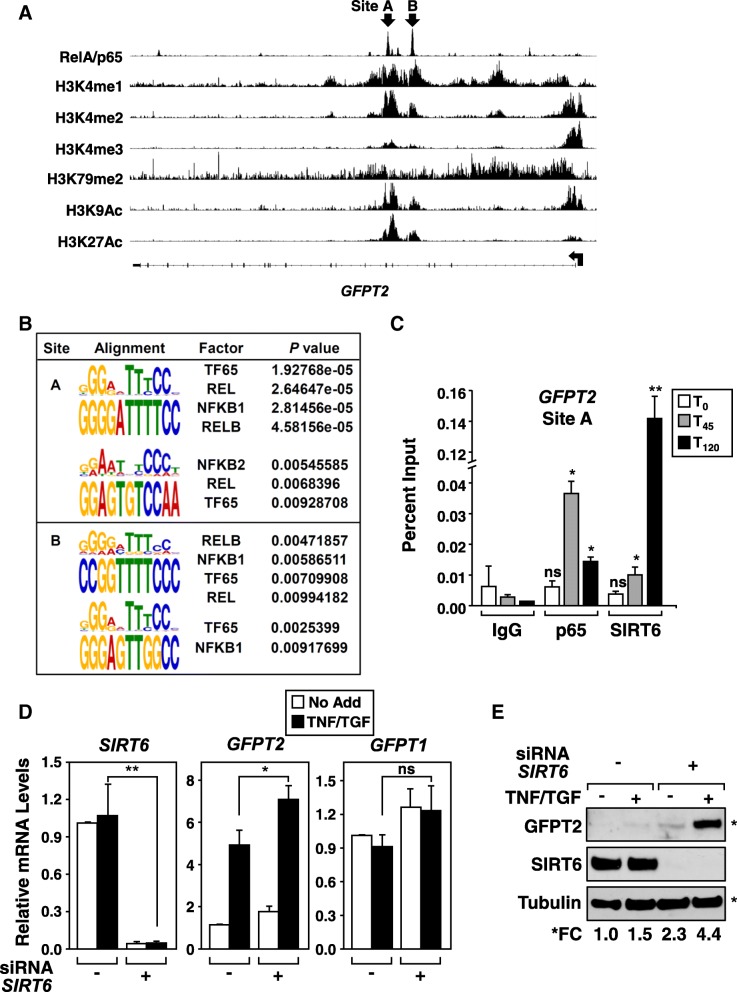
*GFPT2* is a direct target of NF-κB. **a** Screen shot of the *GFPT2* gene showing ChIP-seq enrichments for RelA/p65 (Site A and B) that overlap with histone modifications known to mark transcriptionally active promoters and enhancers. Transcription start site is indicated with an arrow; small rectangles denotes exons across the body of the *GFPT2* gene. **b** MEME Suite and JASPAR were used to analyze sequences contained within sites A and B for NF-κB *cis*-elements. Four of the *cis*-elements had significant *p*-values for NF-κB DNA binding subunits. **c** ChIP-qPCR analysis across the Site A of the *GFPT2* locus indicates elevated chromatin occupancy of p65 and SIRT6 in TNF stimulated A549 cells. Data represent changes in ChIP-qPCR relative to percent input. Mean and SD + are shown; * *p* = < 0.05, ** < 0.01, *n* = 3. **d** Knockdown of SIRT6 in A549 cells increases basal and TNF/TGFβ stimulated *GFPT2* transcripts, while *GFPT1* expression remains unaltered. Changes in mRNA expression were calculated relative to *HPRT* with mean and SD + shown; * *p* = < 0.05, *** < 0.005, ns = not significant, *n* = 3. **e** Immunoblotting of total cell lysates demonstrates that the knockdown of SIRT6 increases basal and cytokine increases in GFPT2 protein expression. Data shown is a representative example, n = 3. Densitometic analysis of immunoblots was used to quantitate fold change of GFPT2 relative to Tubulin

A549 ChIP-Seq datasets were analyzed to examine the chromatin occupancy of RelA/p65 that co-occurred with site-specific histone modifications at the *GFPT2* gene locus following TNF stimulation [30]. ChIP-Seq reads for RelA/p65 showed significant enrichment at two specific peaks (sites A and B), located between intron four and five of the *GFPT2* gene (Fig. 6a). Importantly, the RelA/p65 enrichment sites A and B correlated with histone modifications associated with active enhancers, (histone H3K4me1 and H3K4me2), and with marks known to correlate with active promoters, (H3K4me3, H3K9Ac and H3K27Ac). Both RelA/p65 enrichments and histone H3 modifications overlapped specifically at sites A and B, but were not observed at distal sites surrounding the *GFPT2* proximal promoter (Additional file 1: Figure S3A). Next, sequences contained within sites A and B were analyzed using MEME Suite [33] and motifs were examined using JASPAR [34]. A total of four NF-κB *cis*-elements were identified in sites A and B; each of these elements had significant *p*-values for various NF-κB DNA binding subunits (Fig. 6b).

To confirm that RelA/p65 was directly binding to the *GFPT2* locus we performed quantitative ChIP-PCR analysis on TNF-stimulated A549 cells. Two sets of *GFPT2* primers were used that amplify 200 bp region encompassing either site A or B (, Additional file 1: Table S4). Consistent with ChIP-Seq analysis (Fig. 6a), we observed a time-dependent increase in p65 binding to sites A and B following TNF stimulation, compared to control IgG (Fig. 6c and Additional file 1: Figure S3B). Since Sirtuin 6 (SIRT6) functions as a master regulator of metabolic genes and is known to physically interact with RelA/p65 following TNF-stimulation [19, 39, 40], chromatin occupancy of SIRT6 was also examined. Similar to RelA/p65, SIRT6 bound to *GFPT2* locus at site A and site B with maximum chromatin occupancy observed at 120 min. The chromatin occupancy of SIRT6 at the GFPT2 locus is important, since knockdown of SIRT6 in A549 cells increased basal and TNF-stimulated GFPT2 mRNA and protein expression (Fig. 6d & e). These results indicate that *GFPT2* is a novel NF-κB-regulated gene under the regulation of the NAD^+^-dependent deacetylase SIRT6.

### *GFPT2* is required for mesenchymal cell migration

**Fig. 7:**
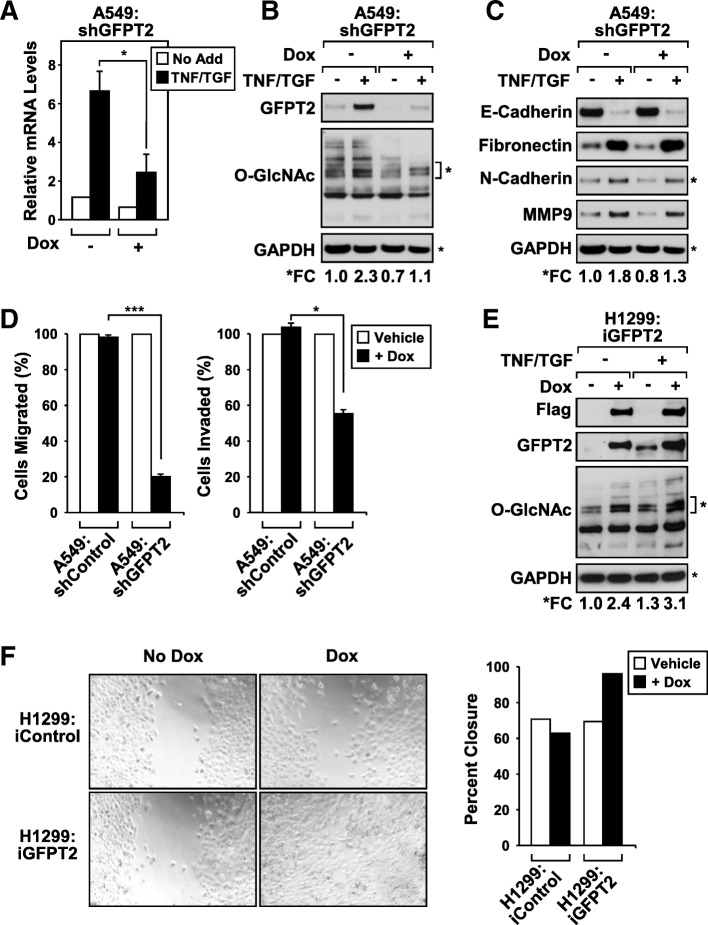
GFPT2 regulates mesenchymal cell migration in NSCLC. **a**-**d** Stable A549 cells expressing doxycycline-inducible shRNA *GFPT2* (A549:shGFPT2) or scrambled control shRNA (A549:shControl) were stimulated with TNF and TGFβ with or without Doxycycline (Dox). **a** Doxycycline treated A549:shGFPT2 cells express significantly lower levels of basal and TNF/TGF-stimulated *GFPT2* transcripts, relative to untreated cells as measured by RT-qPCR. Changes in mRNA expression were calculated relative to *HPRT* with mean and SD + shown; * *p* = < 0.05, ns = not significant, *n* = 3. **b** Knockdown of *GFPT2* dampened TNF/TGF-induced increases in O-GlcNAc modified proteins detected by immunoblotting. Densitometric analysis of the bands indicated (*) relative to GAPDH were used to determine fold changes in O-GlcNAcylated proteins present in extracts after *GFPT2* knockdown. **c** No significant fold change differences in mesenchymal protein marker were detected following *GFPT2* knockdown, as demonstrated by densitometric analysis of the relative N-Cadherin expression compared to GAPDH loading control. **d** GFPT2 expression is required for cell migration and invasion through extracellular matrix as determined in transwell assays. Numbers of migrated and invaded cells without doxycycline treatment for each cell line were considered 100%. Data represents one of three independent experiments, *p = < 0.05, and **p = < 0.01. **e**-**f** Stable H1299 cell line expressing doxycycline-inducible Flag-tagged *GFPT2* (H1299:iGFPT2) or luciferase control (H1299:iControl) were cultured in spheroids with or without TNF and TGFβ stimulation, and with or without the addition of doxycycline. **e** Immunoblot analysis confirms that the inducible expression of GFPT2 increases the abundance of O-GlcNAcylated proteins (*), relative to GAPDH. **f** Light microscopy photographs demonstrate that doxycycline-inducible expression of GFPT2 in H1299 cells increases cell migration as determine in scratch assays. The surface of the gap was measured using TScratch software (45) and data was plotted as percent closure. Data represents one of three independent experiments

To examine the importance of GFPT2 in mesenchymal LUAD, stable A549 cells were generated that expressed *GFPT2* shRNAs (A549:shGFPT2) in a doxycycline-inducible manner. Doxycycline-treated A549:shGFPT2 cells display significantly lower levels of TNF/TGFβ-induced *GFPT2* mRNA expression, compared to cultures left untreated (Fig. 7a). The knockdown of *GFPT2* mRNA expression, correlated with a concomitant loss of GFPT2 protein expression and a decrease in global O-GlcNAc modified proteins (Fig. 7b). Despite this decrease, knockdown of GFPT2 failed to impact mRNA expression of EMT master-switch transcription factors (*SNAI1*, *SNAI2*, and *TWIST1,*, Additional file 1: Figure S4A) and failed to block changes in protein expression of EMT markers, (E-cadherin, fibronectin, N-cadherin, and MMP9, Fig. 7c). Additionally, GFPT2 expression was not required for TNF-induced *BIRC3*/*cIAP-2* and *TNFAIP3*/*A20* mRNA expression, indicating that GFPT2 was not required to potentiate NF-κB transcription (Additional file 1: Figure S4B).

Given that *GFPT2* transcripts are co-expressed along with other important mesenchymal gene signatures (Fig. 3b), we postulated that GFPT2 might be required to regulate cellular phenotypes later in the reprogramming process. To test this, we assayed the migratory properties of A549:shGFPT2 spheroids treated with TNF and TGFβ, compared to A549:shControl cells. Doxycycline-inducible knockdown of GFPT2 significantly inhibited A549 cell migration and dampened cell invasion through Matrigel (Fig. 7d, left and right panels). Results shown in Fig. 7d were not due to off target effects of the shRNA, since similar results were observed in H1299 cells using siRNA-mediated silencing of *GFPT2* (Additional file 1: Figure S4C).

To further examine the importance of GFPT2, we generated stable H1299 cell line expressing doxycycline-inducible V5-tagged GFPT2 protein (H1299:iGFPT2). Doxycycline-induced ectopic expression of GFPT2 exceeded TNF and TGFβ-induced endogenous GFPT2 levels of protein expression (Fig. 7e). Elevated expression of GFPT2 resulted in increased flux through the HBP as determined by an increase in global O-GlcNAcylation (Fig. 7e). Overexpression of GFPT2 led to increased cell migration in H1299:iGFPT2 cells, compared to H1299:iControl cells (Fig. 7f). Together, results shown in Fig. 7 indicate that GFPT2 is both required and sufficient to induce cell motility in mesenchymal NSCLC cells.

### Elevated *GFPT2* expression correlates with poor clinical outcomes in NSCLC

**Fig. 8:**
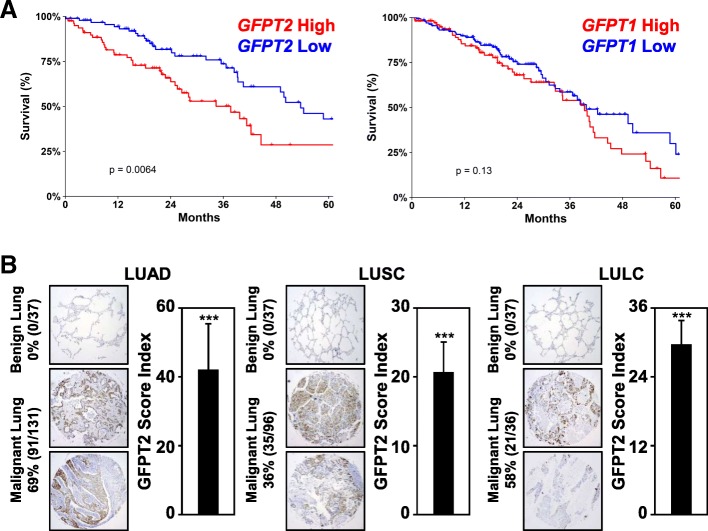
*GFPT2* expression correlates with poor clinical outcomes. **a** Kaplan-Meier analysis using LUAD patients with elevated *GFPT2* mRNA expression show decreased 5-year survival rates, compared *GFPT1* expression which failed to correlate with clinical outcomes. High *GFPT2* expression (> 9 RNA Seq V2 RSEM) verses low (< 6 RNA Seq V2 RSEM); *n* = 517. Median month survival for patients with high *GFPT2* expression 37.68, compared to low *GFPT2* expression 53.61 months. **b** Representative immunohistochemistry images of human lung cancer tissue microarrays stained for GFPT2 expression in malignant lung cancer, compared to adjacent benign lung. The percent of malignant lung cancer that stained positive of GFPT2 is indicated as well as the summary of the overall staining score index, median + SD

Given that GFPT2 promotes motility of lung cancer cells, we examined whether elevated *GFPT2* expression correlated with 5-year overall survival rates in NSCLC. Elevated *GFPT2* mRNA expression correlated with poor clinical outcome in LUAD (*p = 0.0064*, Fig. 8a). Interestingly, *GFPT1 mRNA* expression did not correlate with 5-year overall survival rates (*p = 0.1300*). Elevated *GFPT2* transcripts resulted in a significant decrease in median months survival (MMS, 37.68), compared to cases showing low *GFPT2* expression (MMS, 53.61, Table 2). Moreover, patients with elevated *GFPT2* transcripts also showed a significant decrease in disease free survival compared to LUAD with low *GFPT2* mRNA expression (Additional file 1: Figure S5A). Unlike *GFPT2*, the levels of *GFPT1* mRNA expression did not correlate with overall or disease free survival (Additional file 1: Figure S5B).

**Table 2:** Elevated *GFPT2* mRNA expression correlated with poor clinical outcome in LUAD

Overall Survival			
GFPT2 Expression	Total Cases	Deceased Cases	Median Months Survival
High	79	36	37.68
Low	93	33	53.61

Next, immunohistochemistry was performed on primary LUAD, LUSC and LULC tumors. GFPT2 protein was elevated in 91 out of 131 (69%) LUAD, 35 out of 96 (36%) LUSC, and 21 out of 36 (58%) LULC tumors (Fig. 8b). NSCLC tumors displayed positive staining for GFPT2 in the cytoplasm and at the plasma membrane. While the NSCLC tumors that displayed the highest GFPT2 score indexes were associated with late-stage tumors, the degree of GFPT2 staining by IHC did not correlated with tumor stage in any of the subtypes examined. Collectively, data provided in Fig. 8 indicate that NSCLC tumors display significant upregulation of the rate-limiting enzyme GFPT2, which correlates with overall poor clinical outcomes.

## Discussion

### Identification of GFPT2 as a mesenchymal gene target

High glucose uptake and utilization has been proposed to increase the flux through the HBP in carcinomas [41]. Although increased O-GlcNAcylation has been associated with metastasis, the impact of metabolic reprogramming during EMT is only now beginning to be unveiled [36]. Here, we utilized A549 spheroids cultures stimulated with TNF and TGFβ to identify genes that contributed to metabolic reprogramming. Our studies identified *GFPT2*, the rate limiting enzyme in the HBP pathway, as one of the most highly upregulated gene products in mesenchymal NSCLC cells (Table 1 and Fig. 4c). In support of our study, a report by Lucena et al., found that TGFβ-stimulated A549 NSCLC cells increase glucose uptake and utilization via HBP without global changes in lactate, ATP, pyruvate and glycogen levels [36]. Collectively our findings and the study by Lucena et al. indicate that the induction of EMT upregulates the UDP-GlcNAc nucleotide sugar to coordinate mesenchymal programs. Evidence provided here indicates that *GFPT2* upregulation is co-expressed along with other well-known mesenchymal markers including *SNAI2* and *ZEB2*, and *INHBA* (Fig. 3) [8]. Recently Shaul et al. identified *GFPT2* as a metabolic gene associated with mesenchymal gene signatures in 978 human cancer cell lines [42]. This study supports our findings and suggests that *GFPT2* upregulation is a common EMT gene signature in multiple carcinomas, including LUAD. In our studies, we found that *GFPT2* is transcriptionally upregulated in response to TNF, or combinations of TNF and TGFβ treatments, suggesting that *GFPT2* expression is upregulated in response to inflammatory cytokines present in the tumor microenvironment.

### GFPT2 transcription is upregulated by NF-κB and repressed by SIRT6

Our work identifies *GFPT2* as a novel NF-κB-regulated gene product. We find that RelA/p65 physically interacts with the *GFPT2* gene locus at *cis*-elements located at ChIP-Seq enrichment sites A and B (Fig. 6). These elements are located within intron 4 and 5 contained in the body of the *GFPT2* gene. Consistent with site A and B being important *cis*-regulatory elements for transcriptional activation, we find an enrichment of histone-modifications H3K4me^2^, H3K9Ac and H3K27Ac across these elements. Similar to studies by Martone et al. in which NF-κB has been shown to stimulate gene expression by binding to *cis*-elements located within introns [43], we find that NF-κB binds to enhancer elements located deep within the body of the gene, rather than at proximal upstream promoter domains. However since sites A and B overlap with histone markers associated with transcription, namely H3K4me^2^, H3K9Ac and H3K27Ac, we cannot rule out that NF-κB, along with the proximal promoter, may function as a super-enhancer to drive *GFPT2* transcription in mesenchymal cells.

NF-κB has long been recognized as a transcription factor that regulates metabolic gene products involved in glycolysis, gluconeogenesis, fatty acid biogenesis, and hyaluronan and prostaglandin synthesis. Our laboratory has previously shown that RelA/p65 is a glucose-responsive transcription factor [23]. Since the SIRT6 histone deacetylase is known to regulate metabolic gene targets associated with glucose utilization [39, 44], we examined whether expression of GFPT2 is regulated by SIRT6. In support of SIRT6 regulating the *GFPT2* locus, histone H3K9Ac modification was enriched across site A and B along with RelA/p65 binding (Fig. 6a). Our results demonstrate that SIRT6 is recruited to the *GFPT2* gene locus after RelA/p65 recruitment, a result consistent with other reports indicating that SIRT6 physically interacts with RelA/p65 to repress NF-κB gene targets [19, 40]. Additionally, we find that knockdown of SIRT6 results in an increase in *GFPT2* mRNA and protein levels, indicating that SIRT6 is required to dynamically regulate the *GFPT2* locus in mesenchymal NSCLC.

### GFPT2 is required and sufficient to promote cell migration

Silencing of *GFPT2* in A549 cells dampened detection of O-GlcNAcylated proteins indicating the importance of GFPT2 upregulation to the overall hexosamine biosynthesis following exposure to TNF and TGFβ (Fig. 7). Despite the importance of GFPT2 expression for elevated flux through the HBP, the knockdown of *GFPT2* failed to impact the expression of mesenchymal genes, *SNAI1*, *SNAI2,* and *TWIST1* (Additional file 1: Figure S4A). Additionally, *GFPT2* expression was not required for NF-κB-driven expression of *BIRC3* or *TNFAIP3*. Consistent with these results, the knockdown of *GFPT2* failed to dampen the expression of protein markers of EMT (Fibronectin, N-cadherin, and MMP9) (Fig. 7c). These results were surprising since our laboratory had previously shown that the HBP pathway was critical for the glucose-responsive nature of NF-κB-mediated transcription in response to TNF stimulation [23]. Since we find that the HBP and OGT expression is critical for effective induction of EMT master switch transcription factors and NF-κB activation [23] (Fig. 1), our results suggest that the basal GFPT1 enzyme produces sufficient levels of UDP-GlcNAc to support the induction and maintenance of gene products required for both NF-κB transcription and the induction of the EMT program.

Since EMT-induced activation of the HBP has been shown to increase the expression of cell surface molecules that are heavily glycosylated [36], we examined whether GFPT2 was required for EMT-induced cell migration. Loss of GFPT2 significantly reduced overall A549 cell migration, compared to control cells (Fig. 7). Consistent with the role of GFPT2 in promoting cell motility, doxycycline-inducible expression of GFPT2 in H1299 cells stimulated cell migration, as measured in wound healing assays. Our results indicate that GFPT2 is both necessary and sufficient for migratory properties in mesenchymal NSCLC cells. This is consistent with reports showing that increased glucose flux through the HBP promotes migration and invasion of cholangiocarcinoma cells [45]. Moreover, several groups have reported that hyperglycemic conditions promote migration, invasion and cancer progression of colon cancer [41, 45, 46]. Our work underscores the importance of mesenchymal expression of GFPT2, linking this rate-limiting enzyme with cell migration in NSCLC.

### Elevated GFPT2 expression is associated with poor clinical outcome in NSCLC

Immunohistochemistry analysis demonstrates that LUAD, LUSC, and LULC tumors exhibit elevated GFPT2 protein expression (Fig. 8). While GFPT2 protein levels did not necessarily track with tumor-stage, GFPT2 expression was elevated in all subtypes of NSCLC with the highest incidence found in LUAD and LULC tumors (69 and 58%, respectively). Consistent with immunohistochemistry analysis, elevated *GFPT2* mRNA expression was associated with poor overall survival rates (*p* = 0.0064). Importantly, elevated *GFPT2* mRNA expression, but not *GFPT1*, correlated with reduced median months survival and median months disease-free survival (Table 2 and Additional file 1: Figure S5). The fact that we find that elevated *GFPT2* expression, but not *GFPT1*, correlates with decreased survival rates in LUAD, suggests that the GFPT2 enzyme must serve a unique purpose distinct from GFPT1. GFPT2, unlike GFPT1, is less susceptible to feedback inhibition by its product UDP-GlcNAc [47]. Therefore, it is possible that elevated GFPT2 expression could result in a feedforward production of UDP-GlcNAc to significantly increase the intracellular pool of this nucleotide sugar for post-translational modifications.

GFPT1 and GFPT2 share 80 % amino acid identity between the two enzymes. The largest difference between the two proteins resides in a small twenty-nine amino acid linker region located between the GATase_6 aminotransferase domain and the two SIS phospho-sugar-binding domains. Within this region a proteomic study identified a phosphorylation site at threonine 227 (pT227), [48]. Interestingly, two studies found pT227 GFPT2 in NSCLC. Since T227 is unique to GFPT2, future work will examine whether phosphorylation of this site impacts GFPT dimerization and cellular compartmentalization. Understanding how GFPT2 activity is dysregulated in NSCLC will likely provide insight into ways this metabolic enzyme potentiates cell migration and correlates with poor clinical outcomes in NSCLC.

## Conclusions

This study demonstrates that the rate-limiting metabolic enzyme GFPT2, which is responsible for generation of the nucleotide sugar uridine diphosphate *N*-acetylglucosamine (UDP-GlcNAc), is transcriptionally upregulated by NF-κB and repressed by SIRT6. Modulation of GFPT2 levels alters cell motility and invasion in response to EMT stimuli, affirming its importance in lung cancer progression.

## Additional file

Additional file 1: Is one collated pdf file containing: **Table S1.** shRNA and siRNA sequences. **Table S2.** RT-qPCR primer sequences for *H. sapiens*. **Table S3.** Antibody sources. **Table S4.** ChIP-PCR primer sequences corresponding to the *GFPT2* gene. **Table S5.** Metabolic genes upregulated during EMT. **Figure S1.** RT-qPCR analysis confirms the upregulation of metabolic genes. **Figure S2.** RT-qPCR analysis of GFPT1 in NSCLC following TNF/TGFβ stimulation. **Figure S3.** *GFPT2* is regulated by NF-κB and SIRT6. **Figure S4.** Expression of mesenchymal genes and cell migration following knockdown of GFPT2. **Figure S5.** Overall Survival and Disease-Free Survival. (PDF 711 kb)

## Abbreviations

EMT: Epithelial-to-mesenchymal transition
FN1: Fibronectin 1
GFPT2: Glutamine-fructose-6-phosphate transaminase 2
HBP: Hexosamine biosynthesis pathway
HDAC1, HDAC2, or HDAC3: Histone deacetylases
INHBA: Inhibin, Beta A (Activin A)
LUAD: Lung adenocarcinomas
LULC: Lung large cell carcinoma
LUSC: Squamous cell carcinomas
NF-κB: Nuclear factor kappa B
OGA: O-GlcNAcase
OGT: O-linked N-acetylglucosamine transferase
SIRT6: Sirtuin 6
SR-IκB: Super-repressor IκB
TGFβ: Transforming growth factor beta
TNF: Tumor necrosis factor
UDP-GlcNAc: Uridine diphosphate *N*-acetylglucosamine
VIM: Vimentin
