# Supplementary material for: NF-κB upregulates glutamine-fructose-6-phosphate transaminase 2 to promote migration in non-small cell lung cancer
Source: Cell Commun Signal. 2019 Mar 18;17:24. doi: 10.1186/s12964-019-0335-5 (PMC6421657; doi:10.1186/s12964-019-0335-5)
Supplement: Supplementary file 1 — Is one collated pdf file containing: Table S1. shRNA and siRNA sequences. Table S2. RT-qPCR primer sequences for H. sapiens. Table S3. Antibody sources. Table S4. ChIP-PCR primer sequences corresponding to the GFPT2 gene. Table S5. Metabolic genes upregulated during EMT. Figure S1. RT-qPCR analysis confirms the upregulation of metabolic genes. Figure S2. RT-qPCR analysis of GFPT1 in NSCLC following TNF/TGFβ stimulation. Figure S3. GFPT2 is regulated by NF-κB and SIRT6. Figure S4. Expression of mesenchymal genes and cell migration following knockdown of GFPT2. Figure S5. Overall Survival and Disease-Free Survival. (PDF 711 kb) [file 12964_2019_335_MOESM1_ESM.pdf]

Additional File 1

NF-κB Upregulates Glutamine-Fructose-6-Phosphate Transaminase 2 to Promote Migration in Non-Small Cell Lung Cancer

Szymon J. Szymura[1ψ], Jacob P. Zaemes[1], David F. Allison[1], Sheena H. Clift[1], Jaclyn M. D'Innocenzi[1], Lisa G. Gray[1], Brian D. McKenna[1], Benjamin B. Morris[1,2], Stefan Bekiranov[1], Robin D. LeGallo[2], David R. Jones[3], and Marty W. Mayo[1*]

[1]Department of Biochemistry & Molecular Genetics, [2]Department of Pathology, University of Virginia, P.O. Box 800733, Charlottesville, VA 22908, USA; [3]Department of Thoracic Surgery, Memorial Sloan-Kettering Cancer Center, New York, NY 10065, USA

This document contains the following additional information:

Supplementary Tables (5 total)
Supplementary Figures (5 total)

Supplementary Table 1: shRNA and siRNA sequences

Company	Target	siRNA Target Sequence
GE Dharmacon<br>(D-001206-13)	siRNA Non-targeting Control<br>Pool 1	UAGCGACUAAACACAUCAA<br>UAAGGCUAUGAAGAGAUAC<br>AUGUAUUGGCCUGUAUUAG<br>AUGAACGUGAAUUGCUCAA
GE Dharmacon<br>(M-019111-00)	Human *OGT*<br>SMART pool	GCAGUUCGCUUGUAUCGUA<br>CGACAUGCAUUGCGUCUCA<br>GAUUAAGCCUGUUGAAGUC<br>CCGAGAAAUUGGCUUAUAU
GE Dharmacon<br>(M-010390-01)	Human *GFPT2*<br>SMART pool	UCGCCAAGCUGAUUAAAUA<br>GAUCAUCCGUGGCUUGAGA<br>AGACAAAGGCAACGAAUUU<br>CGAUGGGAAUAAUCACGAA

Company	Target	Mature Target Sequence
GE Dharmacon<br>(V2THS_50314)	TRIPZ human GFPT2 shRNA	TCAGATGAAAGGTGGTGAT

Supplementary Table 2: RT-qPCR primer sequences

Primer Sequences for *H. sapiens*

**Gene**	**Sequence**
GAPDH F	GAAGGTGAAGGTCGGAGTC
GAPDH R	GAAGATGGTGATGGGATTTC
SNAI1 F	CACTATGCCGCGCTCTTTC
SNAI1 R	GGTCGTAGGGCTGCTGGAA
SNAI2 F	ATGAGGAATCTGGCTGCTGT
SNAI2 R	CAGGAGAAAATGCCTTTGGA
ZEB2 F	CAATACCGTCATCCTCAGCA
ZEB2 F	CCAATCCCAGGAGGAAAAAC
TWIST1 F	CGGGAGTCCGCAGTCTTA
TWIST1 R	CTTGAGGGTCTGAATCTTGCT
OGT F	AGGAAATGTCTTGAAAGAGGCAC
OGT R	TCGTAGTACACACAAGCCCAGG
SLC2A1 F	CATCAACGCTGTCTTCTATTACTC
SLC2A1 R	ATGCTCAGATAGGACATCCA
SLC1A4 F	CTGTGGACTGGATTGTGGAC
SLC1A4 R	TCCACTTTCACCTCAGCAAG
NT5E F	TGGAGATGGGTTCCAGATGATAA
NT5E R	GGATAAATTACTTTCATTTTGGAGATAT
UUP1 F	ACTGCCCAGGTAGAGACTATC
UUP1 R	CTGCACCAGCTTCTTGTTAAG
GFPT1 F	CCAGCCAGTTTGTATCCCTT
GFPT1 R	CAAGCATGATCTCTTTGCGT
GFPT2 F	AGGTGCATTCGCGCTGGTT
GFPT2 R	TGTGGAGAGCTTGTATTTGCTCCCGGG
UAP1 F	AATGACCTCAAACTCACGTTGT
UAP1 R	GCTCTGCATAAAGTTCTACCTGT
MGEA5 F	TCCATAACCCAAGGTCTTCCA
MGEA5 R	TTGGAGGAGCGGGAGAGCGA
TNFAIP3 F	TTGACCAGGACTTGGGACTT
TNFAIP3 R	ACAGCTTTCCGCATATTGCT
BIRC3 F	GCTGTGATGGTGGACTCAGG
BIRC3 R	TGGCTTGAACTTGACGGATG
HPRT F	TTGGAAAGGGTTATTCCTCA
HPRT R	TCCAGCAGCTCAGCAAAGAA

Supplementary Table 3: Antibodies

Antibody Target	Company	Product Number
α-Tubulin	Sigma	T6793
E-Cadherin	Santa Cruz	sc-7870
Fibronectin	BD Bioscience	610078
Flag M2	Sigma	F1804
GAPDH	GeneTex	GTX627408
GFPT1	Cell Signaling	3818
GFPT2	Cell Signaling	6917
GFPT2 (IHC)	Atlas	HPA059910
MMP9	R&D Systems	MAB936
N-Cadherin	BD Transduction Labs	610920
Normal IgG (ChIP)	Santa Cruz	sc-2025 (mouse) Rabbit sc-2027
OGA	Sigma	SAB4200311
OGT	Sigma	O6264
O-GlcNAc RL-2	Thermo Scientific	MA1-072
O-GlcNAc CTD10.1	Cell Signaling	98705s
p65 C-20 (ChIP)	Santa Cruz	Sc-372
V5	Invitrogen	R960-25
Vimentin	Sigma	V6630

Supplementary Table 4: ChIP-PCR primer sequences corresponding to the *GFPT2* gene

***GFPT2* Locus**	**Sequence**
H. sapiens ChIP site A F	TCCTGCCCTTCCCAGTGATAAA
H. sapiens ChIP site A R	CTTTGCTTATTGTCCTGTTG
H. sapiens ChIP site B F	AGTGGCAAGGGTGAGCTTCT
H. sapiens ChIP site B R	TTGCACAATGCCTGCCTG

Site A:

TCCTGCCCTTCCCAGTGATAAAATGAACCTCATT**GGAAAATCCC**CCACTTCAGTGTAGATGATGATTCTCATTG
TGTCCCCAAAGCAGGGAGGCTCCCAGGAGGCCCACCCACACCCAAACT**TGGACACTCC**AGCCCTGACTCAGGAT
GAGTAATTGAAAAGAAAACAACAGGACAATAAGCAAAG

Site B:

AGTGGCAAGGGTGAGCTTCTGAGGTGGGATGATCTCAGGGCCTCCGGGAGTGACTCAGAGGTATTTGGCTGTGA
TGCTGGCAAACATTTTTCCCCCCTTC**CCGGTTTTCCC**TGTTCTTGACTTAAGTCACTCCAAGGGCAGATCACCA
GGGAGGAGA**GGGAGTTGGCC**AGGCAGGCATTGTGCAA

SUPPLEMEMTARY TABLES

Supplementary Table 5:
Metabolic genes upregulated during EMT

**Genes**		
ABAT	GLS1	SLC2A1
ACO1	GPAM	SLC4A7
ACSL5	HK2	SLC6A6
ADA	HKDC1	SLC6A8
AGPAT4	HLCS	SLC6A10P
AGPAT6	HMOX2	SLC16A2
AK8	HS3ST3B1	SLC22A4
ATP5C1	IDS	SLC25A37
B4GALT1	INPP4B	SLC39A8
BCAT1	ITPKC	SLC43A3
BMP2	LSS	SMAD5
BPGM	MAP2K1	SMAD7
CDA	MBOAT2	SMOX
CHKB	MTAP	SMS
CHST11	MTHFD1L	SOD2
CHST2	NAMPT	SPHK1
CPT1B	NDST1	SPTLC2
CYP2B6	NEDD4L	SQLE
CYP2R1	NT5E	ST3GAL1
DHRS3	ODC1	SYNJ2
DIO2	PANK2	TDO2
DSE	PECR	UAP1
ENO3	PGK2	UBA6
FA2H	PGM2	UBE2D1
FSTL1	PIK3CD	UBE2D3
GAD1	PLCB4	UBE2E2
GALNT12	PLD1	UBE2O
GALNT5	PNPLA8	UPP1
GCH1	PPAP2B	UXS1
GFPT2	PTGES	XDH
GGH	PTGS2	XYLT1
GK	PYGL	
GK3P	SAT1	

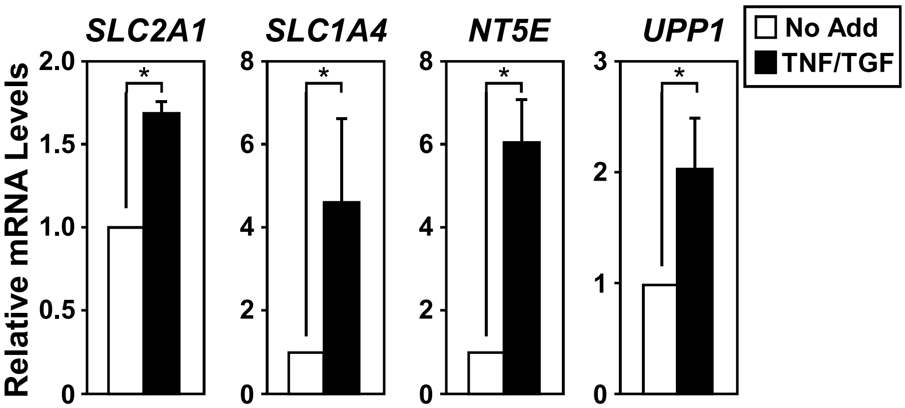

Supplementary Figure 1: RT-qPCR analysis confirms the upregulation of metabolic genes. Data represents three independent experiments performed in triplicate; mRNA levels were calculated relative to *HPRT* mRNA expression. Mean +SD are shown; * p= < 0.05.

Szymura *et al.*
Supplementary Figure 2

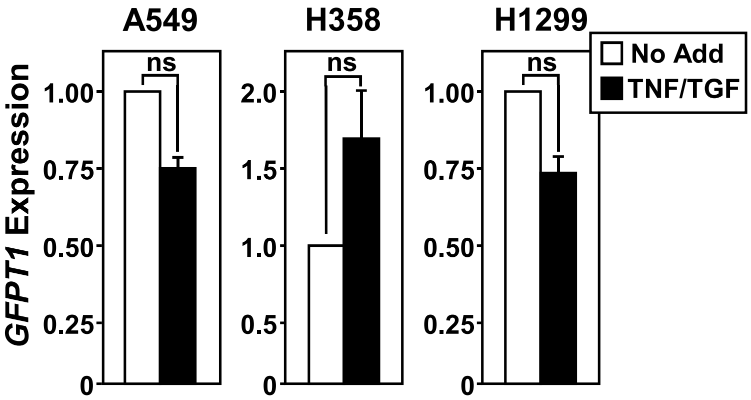

Supplementary Figure 2: Unlike *GFPT2, GFPT1* mRNA expression is not upregulated in NSCLC spheroid cultures following TNF and TGFβ stimulation, as detected by RT-qPCR analysis. Data represents three independent experiments performed in triplicate; mRNA levels were calculated relative to *HPRT* mRNA expression. Mean ± SD are shown; ns – not significant.

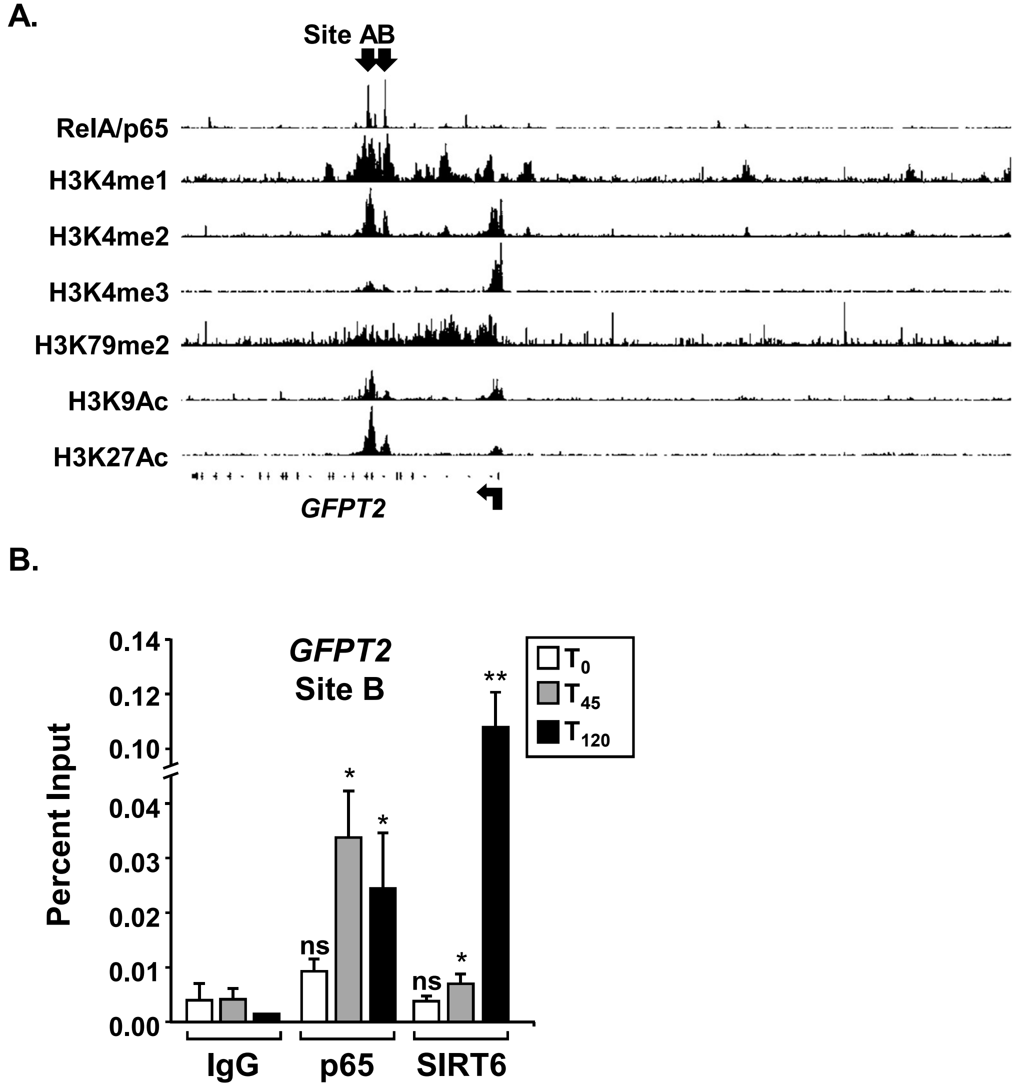

Supplementary Figure 3: The *GFPT2* gene is regulated by NF-κB and SIRT6. (A) A screen shot of the *GFPT2* gene showing ChIP-seq enrichments for RelA/p65 are localized to Site A and B, which correspond to the image shown in Figure 6A. (B) ChIP-qPCR analysis across the Site B of the *GFPT2* locus indicates elevated chromatin occupancy of p65 and SIRT6 in TNF stimulated A549 cells. Data represent changes in ChIP-qPCR relative to percent input. Mean and SD ± are shown; * p = < 0.05, ** < 0.01, ns - not significant; n = 3.

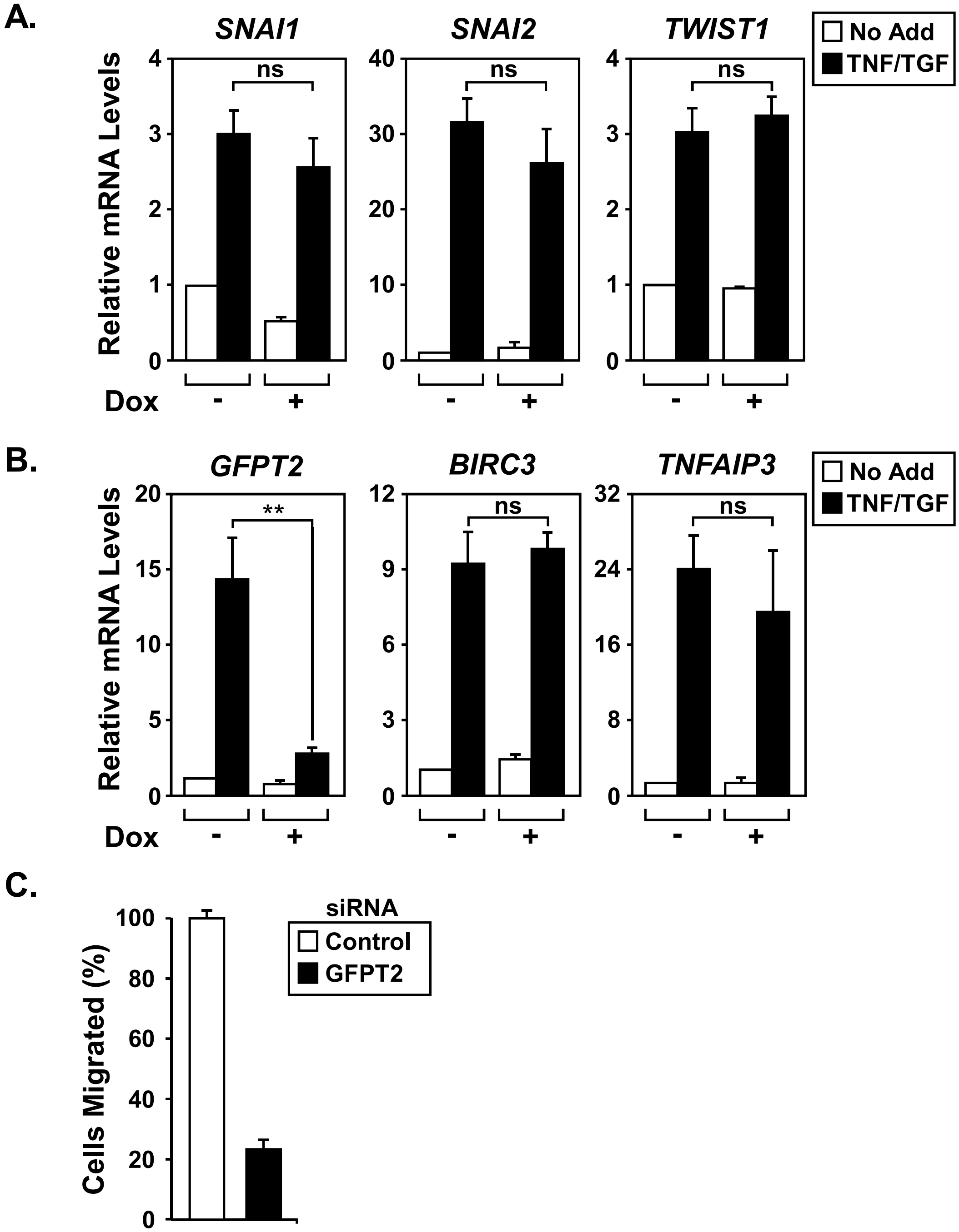

Supplementary Figure 4: Knockdown of *GFPT2* fails to impact the expression of NF-κB-regulated genes as well as mesenchymal markers, but is required for cell migration. (A & B) Knockdown of GFPT2 following Dox treatment of A549:shGFPT2 cells fails to show changes in mesenchymal markers *SNAI1*, *SNAI2*, or *TWIST1,* as well as NF-κB transcriptional targets, *BIRC3* and *TNFAIP3* following stimulation. (C) siRNA-mediated silencing of GFPT2 abolished H1299 cell migration, in a similar manner as data shown in Figure 7D. Mean and SD ± are shown; ** p = < 0.01, ns - not significant; n = 3.

A.

Disease Free Survival

GFPT2 Expression	Total Cases	Relapsed/Progressed Cases	Median Months Survival
High	63	36	22.9
Low	82	34	44.02

B.

Overall Survival

GFPT1 Expression	Total Cases	Deceased Cases	Median Months Survival
High	101	44	39.32
Low	133	45	39.72

Disease Free Survival

GFPT1 Expression	Total Cases	Relapsed/Progressed Cases	Median Months Survival
High	82	39	26.94
Low	118	52	28.42

Supplementary Figure 5: Elevated *GFPT2*, but not *GFPT1*, mRNA expression correlates with poor overall and disease free survival.(A) As shown in Table 3 elevated *GFPT2*expression is associated with reduced overall survival and with reduced median months disease free survival. (B) Unlike *GFPT2*, *GFPT1*expression is not predictive of overall or disease free survival.
